# Cytotoxic Aggregation and Amyloid Formation by the Myostatin Precursor Protein

**DOI:** 10.1371/journal.pone.0009170

**Published:** 2010-02-11

**Authors:** Carlene S. Starck, Andrew J. Sutherland-Smith

**Affiliations:** Institute of Molecular BioSciences, Massey University, Palmerston North, New Zealand; Monash University, Australia

## Abstract

Myostatin, a negative regulator of muscle growth, has been implicated in sporadic inclusion body myositis (sIBM). sIBM is the most common age-related muscle-wastage disease with a pathogenesis similar to that of amyloid disorders such as Alzheimer's and Parkinson's diseases. Myostatin precursor protein (MstnPP) has been shown to associate with large molecular weight filamentous inclusions containing the Alzheimer's amyloid beta peptide in sIBM tissue, and MstnPP is upregulated following ER stress. The mechanism for how MstnPP contributes to disease pathogenesis is unknown. Here, we show for the first time that MstnPP is capable of forming amyloid fibrils *in vitro*. When MstnPP-containing *Escherichia coli* inclusion bodies are refolded and purified, a proportion of MstnPP spontaneously misfolds into amyloid-like aggregates as characterised by electron microscopy and binding of the amyloid-specific dye thioflavin T. When subjected to a slightly acidic pH and elevated temperature, the aggregates form straight and unbranched amyloid fibrils 15 nm in diameter and also exhibit higher order amyloid structures. Circular dichroism spectroscopy reveals that the amyloid fibrils are dominated by β-sheet and that their formation occurs via a conformational change that occurs at a physiologically relevant temperature. Importantly, MstnPP aggregates and protofibrils have a negative effect on the viability of myoblasts. These novel results show that the myostatin precursor protein is capable of forming amyloid structures *in vitro* with implications for a role in sIBM pathogenesis.

## Introduction

Myostatin is a member of the transforming growth factor-beta (TGF-β) superfamily of growth and differentiation factors and is a primary regulator of muscle growth both pre- and postnatally, primarily via inhibition of myoblast proliferation and differentiation [1]–[4]. Like other members of the family, myostatin is translated as a precursor protein (MstnPP) that consists of an N-terminal signal sequence, a regulatory propeptide domain (residues 21–266) and a growth factor domain (residues 267–374) which dimerises at the C-terminus via an inter-molecular disulfide bond [5]–[7]. The mature growth factor dimer is cleaved from the propeptide region by furin convertase proteolysis in the endoplasmic reticulum (ER) at a conserved RSRR sequence [2], [7]. The propeptide region plays at least two important functions. First, as a chaperone in the ER to assist in the folding of the growth factor region [8], [9] that contains the intricate cysteine-knot motif characteristic of the family [10], [11]. Second, the N-terminal propeptide plays a regulatory role after cleavage, remaining non-covalently associated with the mature dimer to form a latent complex which is exported from the cell [7]. Myostatin remains latent until a second cleavage event immediately N-terminal to aspartate 76 of the propeptide region, most probably by metalloproteinases, that disrupts the association [12], [13]. It is possible that furin cleavage of MstnPP also occurs post-secretion with a pool of extracellular MstnPP identified in skeletal muscle [14]. The mature growth factor dimer is structurally similar to other members of the TGF-β family [11]; however the structural characteristics of MstnPP or the propeptide region remain undetermined.

Signalling by the myostatin growth factor via activin and TGF-β receptors [2] ultimately results in cell-cycle arrest through the upregulation of genes involved in cell-cycle withdrawal such as p21 and p53 and the downregulation of myogenic regulatory factors such as MyoD and myogenin [15]–[18]. Although postnatally this action maintains the quiescence of muscle satellite cells, the prenatal role is more complex and depends on the environmental context during development, with signalling by myostatin ensuring that a balance between proliferation and differentiation is maintained [19].

Myostatin-null mutations have been identified in dogs, cattle and sheep, resulting in a double-muscled phenotype [1], [20], [21] and an exceptionally muscular and strong human lacking functional myostatin protein was also recently identified [22], [23]. Myostatin overexpression in mice induces profound muscle and fat loss analogous to that seen in human cachexia syndromes [4] and ectopically expressed myostatin rapidly lowers muscle mass in rats [24]. Myostatin signalling can have negative consequences in a diseased background such as the muscular dystrophies [25] and may contribute to cachexia associated with many chronic disease states [4] including HIV [26] and cancer [27]. For these reasons, since its discovery in 1997 [3], the processed myostatin growth factor dimer has been suggested to hold exciting potential for inhibitory targeting in a wide range of muscle wastage diseases [28], [29].

Less focus had been placed on the involvement of MstnPP or the propeptide region in disease until a role for MstnPP in the pathogenesis of sporadic inclusion body myositis (sIBM) was proposed [30], [31]. sIBM is the most common progressive muscle wastage disease associated with aging where progressive muscle loss leads to severe atrophy and weakness. Although the pathogenesis is unknown, it is likely that oxidative damage contributes to aging of the muscle fibers [30]. Endoplasmic reticulum (ER) stress and the unfolded protein response (UPR) have been demonstrated in sIBM muscle fibers [30], [32]. Inflammation and amyloid formation appear to be predominant features but whether these are causally related and which is the primary cause of sIBM, remain matters of debate [30], [33]. The presence of fibrillar inclusions in some diseased tissue suggests that sIBM may be an amyloid disease, where a prominent feature is protein aggregation and the subsequent formation and deposition of large amyloid fibrils analogous to those observed for neurodegenerative disorders such as Alzheimer's, Parkinson's and Huntington's diseases, the spongiform encephalopathies, the systemic amyloidoses and type II diabetes [34], [35]. Despite extensive research, the mechanisms behind amyloid formation and how they contribute to disease remain poorly understood. It is well-documented that fibril formation occurs via one or more oligomeric intermediate stages [36]–[40] and it appears that an intermediate oligomeric species, rather than the mature fibril, is the mediator of cyto- and neurotoxicity [41]–[43]. Amyloid fibrils, characterised by extensive β-sheet secondary structure, are deposited in tissues and are resistant to degradation but may actually be chemically inert [35].

The filamentous inclusions of sIBM tissue contain a number of molecules normally alien to muscle fibers, predominantly the amyloid beta (Aβ) protein of Alzheimer's disease [44]. Biopsies from sIBM muscle fibers revealed a close association of the myostatin precursor protein (MstnPP) with Aβ aggregates and increased levels of both MstnPP and the processed myostatin growth factor dimer [31]. Furthermore, when muscle cells are placed in conditions that result in endoplasmic reticulum (ER) stress, MstnPP is upregulated and an sIBM-like pathology is observed [45]. ER stress may lead to the misfolding and aggregation of MstnPP. Cultured human muscle fibers (CHMFs) overexpressing Aβ precursor protein showed an increase in MstnPP protein levels and subsequent experimental inhibition of the proteasome caused co-accumulation of MstnPP and AβPP/Aβ within aggresomes [46]. CHMFs subjected to tunicamycin or thapsigargin, compounds that inhibit protein glycosylation and disrupt ER calcium levels respectively inducing ER stress, also showed an increase in MstnPP expression (in addition to ER stress marker proteins GRP78 and Herp), possibly due to the activation of NF-κB [45]. In contrast to proteasome inhibition, where MstnPP mRNA levels were decreased, NF-κB activation increases transcription of MstnPP mRNA in a stressed situation. These differences may be explained by the observation that the nature and duration of ER stress may be important in determining whether a cell activates a protective pathway or enters apoptosis [47], [48]. An additional mechanism for how myostatin/MstnPP contributes to sIBM is focused on increased activity by the myostatin growth factor. As myostatin is a procachectic growth factor postnatally, increased production and processing of MstnPP may result in increased levels of circulating myostatin growth factor and signalling that leads to atrophy [45], [46], [49], though there is little evidence supporting this hypothesis. A number of peptide hormones, such as those in secretory granules of the endocrine system, have been found to aggregate into amyloids in secretory pathways in a regulated, functional manner [50], [51], so there is a possibility that myostatin aggregates are protective in the sIBM diseased state.

The role of MstnPP in sIBM has not been firmly established. Our research shows for the first time that MstnPP is able to form amyloid fibrils independently *in vitro* via a pathway that produces cytotoxic intermediates. When MstnPP is refolded and purified from *E. coli* inclusion bodies, a population of MstnPP spontaneously misfolds into amyloid protofibril-like aggregates that can be visualised using electron microscopy and bind the amyloid-specific dye thioflavin T. When subjected to a slightly acidic pH and elevated temperature, the protofibril-like aggregates form straight and unbranched amyloid fibrils that are 15 nm in diameter and exhibit higher order amyloid structures such as twists and bundles. The fibrils are resistant to trypsin digest after incubation overnight at 37°C. Circular dichroism spectroscopy reveals that MstnPP undergoes a number of structural transitions through aggregation to amyloid protofibril and fibril formation that initiate at a physiologically relevant temperature. Significantly, MstnPP aggregates and protofibrils have a negative effect on the viability of C2C12 mouse myoblasts when added to the culture medium. These results support a role for MstnPP in the pathogenesis of sIBM and present the hypothesis that this role may be within a degenerative context.

## Results

### MstnPP Is Predicted to Be Prone to Aggregation and Disorder *In Silico*

**Figure 1:**
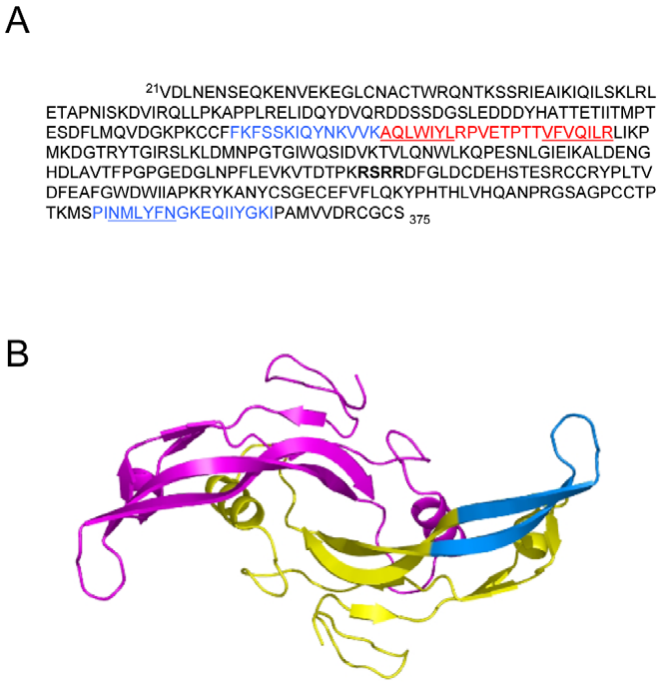
*In silico* predictions of propensity for β-sheet aggregation (Tango and PASTA) and amyloid formation (Waltz). A. MstnPP sequence 21–375 showing regions of elevated propensity at two levels; red regions have higher propensity than those in blue. Regions where Waltz, Tango and PASTA predictions overlap are underlined. The RSRR furin proteolysis sequence that separates the propeptide from the growth factor domain is shown in bold. B. Mapping the 348–364 sequence (blue) onto the myostatin growth factor crystal structure. The region has been shown on one monomer only.

*In silico* programs have been shown to accurately predict the regions of a polypeptide chain that are prone to β-sheet aggregation and amyloid formation. The MstnPP amino acid sequence was analysed for β-aggregation propensity (Fig. 1A) using Tango [52] (Fig. S1A) and PASTA [53], [54] (Fig. S1B) and for amyloidogenic regions using Waltz [55] (Fig. S1C). Both Tango and PASTA predicted a high propensity for β-aggregation for residues within the pro-peptide region, 154–177 (Fig. 1A); residues 139–153 were also predicted by PASTA to have β-aggregation tendencies, albeit at a comparatively lower level. Tango also identified growth factor domain sequences, 314–321 and 347–355. Waltz predicted the same propeptide amyloidogenic regions, 153–162 and 168–177 as well as the very C-terminus of the protein, 348–364. Mapping of aggregation-prone regions of the myostatin growth factor onto the published crystal structure [11] shows that residues 348–364 localise to the β-sheet ‘fingers’ of the growth factor (Fig. 1B).

### *In Vitro* Refolding of MstnPP Results in the Production of Large Soluble Aggregates

**Figure 2:**
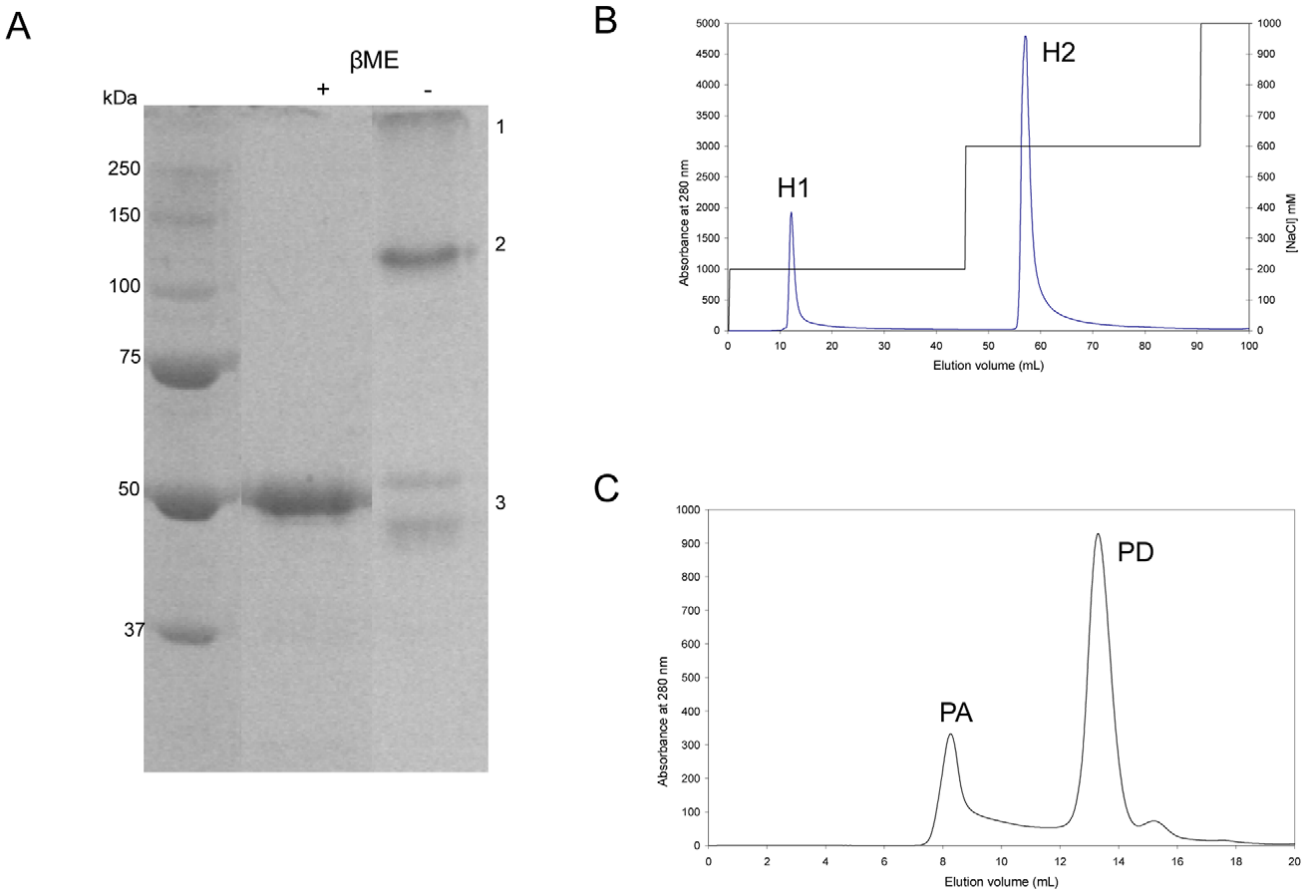
Refolding and purification of human MstnPP. A. Reducing (+ β-mercaptoethanol (βME)) vs non-reducing (−βME) SDS-PAGE (12%) after refolding. β-ME concentration is 2 M. Bands are as indicated: 1. soluble aggregates; 2. disulfide-bonded dimer; 3. monomer either fully reduced (+β-ME) or misfolded at various stages of reduction (−β-ME). Figure assembled from one gel where intervening lanes were removed. B. Heparin affinity chromatography separates the majority of soluble aggregates (H2) from correctly-folded dimer (H1). C. S200 gel filtration chromatography; the MstnPP dimer elutes at 13.5 mL (PD) and the soluble aggregates in the void volume at 8 mL (PA).

Human MstnPP is found in inclusion bodies when overexpressed in *E. coli* like other TGF-β family members [56], [57] and can be refolded *in vitro* using a modification of methods described for zebra-fish myostatin [8]. After refolding, in addition to the native disulfide-bonded MstnPP dimer, two other species were apparent by non-reducing SDS-PAGE; misfolded monomer and a very high molecular weight species that has been characterised previously as soluble aggregates [8], [9] (Fig. 2A), which are frequently observed during protein refolding [58]. Native MstnPP was purified by heparin affinity chromatography that separated the correctly folded MstnPP dimer from the majority of the aggregates followed by the removal of remaining aggregates by gel filtration. During heparin chromatography, the correctly folded dimer eluted at 200 mM NaCl (H1) and the majority of aggregates as a large peak at 600 mM NaCl (H2) (Fig. 2B and Fig. S2A). Western blotting identifies both peaks as myostatin (Fig. S2B) but the two species interact differently with the heparin column suggesting a difference in folding. Further purification of both peaks was achieved using gel filtration (Fig. 2C). The aggregates are too large to move from the well into an SDS-PAGE gel (above 250 kDa, Fig. 2A), elute in the void volume during S200 gel filtration (PA) (Fig. 2C) and remain soluble even after ultracentrifugation at 214,000×g (data not shown). Since the MstnPP monomer is 43 kDa the soluble aggregates are most likely to be an oligomer of at least 5 monomeric units.

### MstnPP Soluble Aggregates Have Characteristics of Amyloid-Like Protofibrils

**Figure 3:**
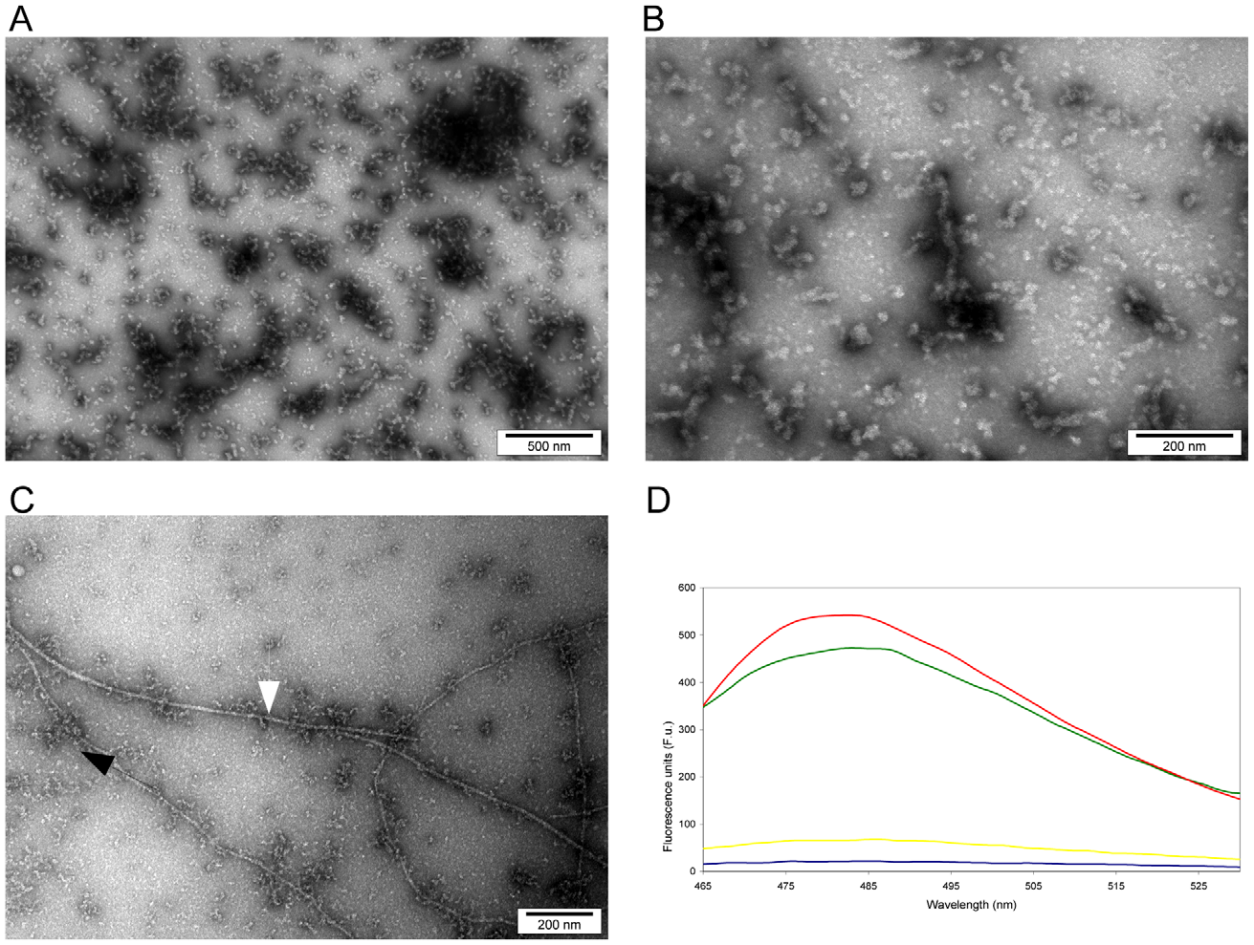
Characterisation of MstnPP soluble aggregates by negative-stain transmission electron microscopy (TEM) and ThT binding. (A–B) TEM of MstnPP soluble aggregates and C. insulin positive control containing both protofibrils (black arrow) and mature fibrils (white arrow). D. ThT binding of MstnPP soluble aggregates (green), compared to an insulin positive control (red), the MstnPP dimer (yellow) and a buffer (50 mM Tris-HCl pH 8.5, 150 mM NaCl) blank (blue).

Transmission electron microscopy (TEM, Fig. 3A and B) revealed that MstnPP soluble aggregates exhibit a morphology and size similar to that documented for amyloid protofibrils from a number of other proteins such as lysozyme [59], [60] and HypF-N [61] as well as the insulin protofibrils generated as a positive control (Fig. 3C) using a well-established protocol [39], [62], [63]. A range of structures, including spheres and oligomers of associated spheres (diameter 15.6±1.6 nm SD), can be seen, and examples of increased elongation of the aggregates are apparent (Fig. 3B).

A defining characteristic of amyloid fibrils and protofibrils is their ability to bind the fluorescent dye thioflavin T (ThT) [63]–[65]. When bound to fibrils, ThT can be excited at 450 nm to fluoresce at 485 nm [66]. One limitation of this method is the lack of a strict quantitative relationship [64] with amyloid fibrils often producing a stronger emission than protofibrils by comparison [65]. ThT binding assays were carried out on the MstnPP aggregates and compared to a solution containing a mixture of insulin protofibrils and fibrils as a positive control. The MstnPP soluble aggregates bound ThT with intensity comparable to insulin fibrils whereas the correctly folded MstnPP dimer did not (Fig. 3D). These results suggest that a population of MstnPP can aggregate spontaneously to form amyloid-like protofibrils.

### Formation of MstnPP Amyloid Fibrils at Acidic pH and Elevated Temperature

**Figure 4:**
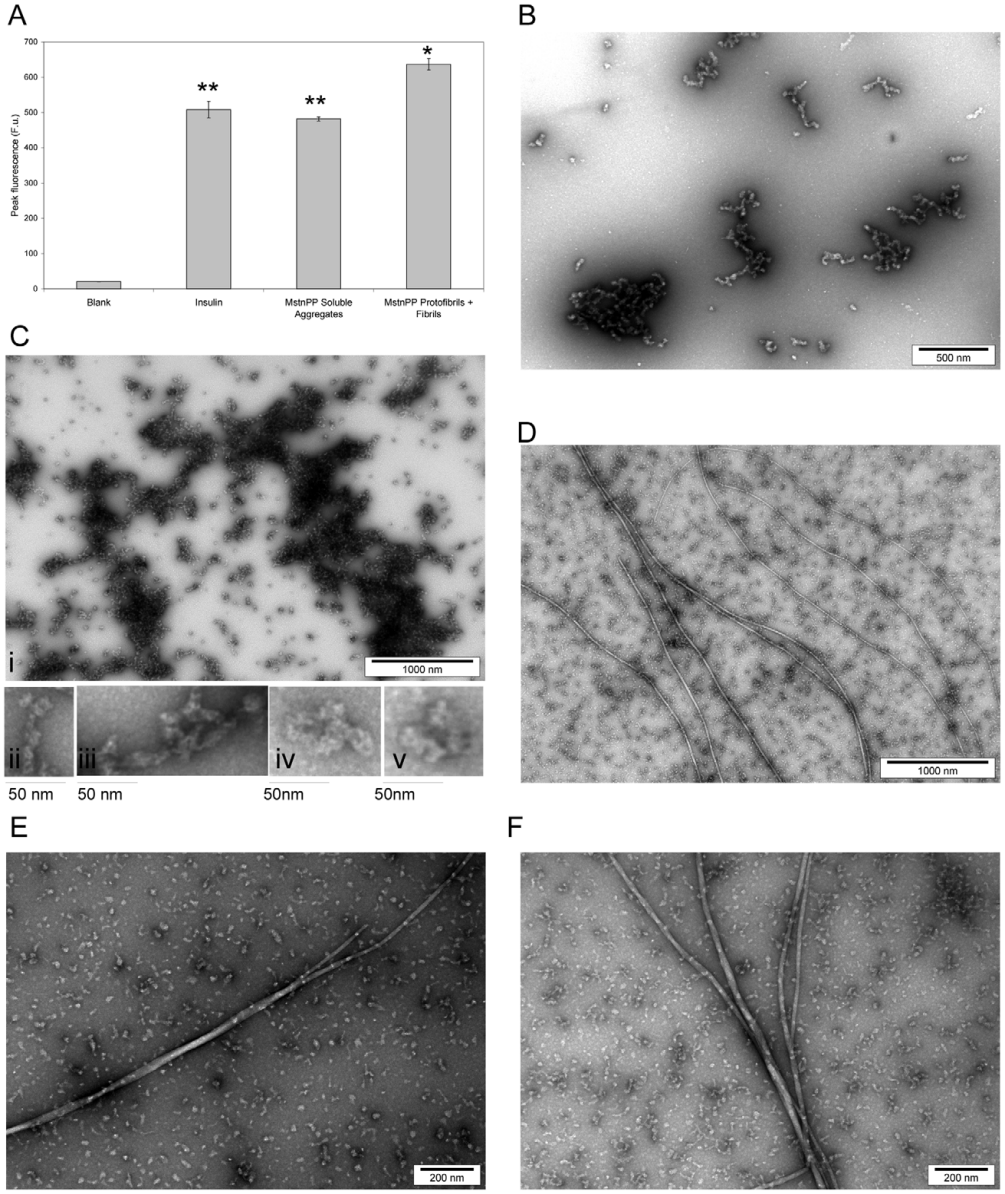
Characterisation of MstnPP amyloid fibrils by ThT binding and TEM. A. ThT binding after incubation at 60°C and pH 5.3 compared to insulin and MstnPP soluble aggregates. Each result represents the average of four independent experiments and error bars represent SEM. ** P<0.005 and * P<0.01 by paired Student's t-test. Blank is 0.005 mM HCl, pH 5.3 and ThT. (B–F) TEM showing different stages in the formation of prefibrillar structures and fibrils by human MstnPP. B. Overnight incubation at 60°C in pH 5.3 produces ‘beaded’ prefibrillar structures; C. (i) By three days large, dense three-dimensional arrays show up as areas of high electron density, (ii–v) zoomed in examples of lateral association and longitudinal fusion (ii and iii) and pore-like structures (iv and v) from (i); D. Characteristic amyloid fibrils after one week; E–F. Formation of higher order structures with the twisting of two (E) or more (F) fibrils around each other.

The presence of MstnPP amyloid-like protofibrils suggests that amyloid fibril formation is also possible. MstnPP aggregates were concentrated and resuspended in dilute HCl solutions (pH range 1.6 to 6.3) and incubated at either 37° or 60°C, conditions including those under which insulin forms amyloid fibrils [62], [63]. Solutions were monitored by ThT binding and TEM (Fig. 4). After one week at 60°C, ThT fluorescence had increased significantly in the pH 5.3 solution (Fig. 4A), which contained a mixture of prefibrillar aggregates and amyloid fibrils observed by TEM (Fig. 4B–4F) in both the presence and absence of 0.1% sodium azide. The fluorescence assays were also performed by diluting protein solutions into a pH 7.5 buffer since ThT binding may be affected by changes in pH [67]. In these assays, fluorescence values for the soluble aggregates were largely unchanged but an increase in intensity for the pH 5.3 samples was observed (data not shown), consistent with the lower pH results.

Myostatin fibril formation occurs via a number of stages. For a 3 mg/mL solution, overnight incubation at 60°C in pH 5.3 produces oligomers that are compact and have a ‘bead-on-a-string’ morphology (Fig. 4B) as observed for Aβ and lysozyme fibrils [60], [65]. By three days, extensive arrays of aggregation were present; these are often three-dimensional, appearing as areas of high electron density under the TEM (Fig. 4Ci). Increased elongation and in some places lateral association and longitudinal fusion of oligomers can be observed (Fig. 4Cii and 4Ciii). This granular to smooth transition has been shown during Aβ fibril formation [65]. Smaller pore-like oligomers, previously documented on the amyloid formation pathway of a number of proteins [40], [43], are also apparent (Fig. 4Civ and 4Cv). By one week, fibrils that show the characteristic morphology of amyloid had appeared, with a diameter of 15.4±0.7 nm (SD) and a straight, unbranching structure (Fig. 4D). The fibrils are extremely long, in excess of 5 µm. At two weeks, higher order amyloid structures can be observed, such as two (Fig. 4E) or more (Fig. 4F) fibrils twisting around each other.

### Secondary Structure Analysis of MstnPP Fibril Formation by Circular Dichroism Spectroscopy

**Figure 5:**
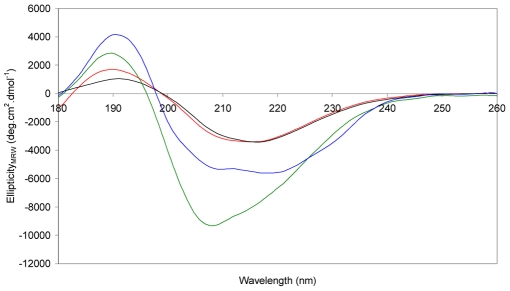
Circular dichroism spectra showing the structural transitions that occur in amyloid fibril formation. Correctly folded MstnPP dimer (blue); MstnPP soluble aggregates before acidification (green); prefibrillar aggregates after overnight incubation in pH 5.3 at 60°C (red); mixture of prefibrillar aggregates and fibrils after one week incubation (black).

Amyloid formation is accompanied by the adoption of a β-sheet rich secondary structure regardless of the structure of the native protein [68]. Circular dichroism (CD) spectroscopy was used to study the structural changes that occur during amyloid formation (Fig. 5). Correctly refolded MstnPP has a CD spectrum indicative of a mixture of α-helix and β-sheet, in agreement with the crystal structure of the mature myostatin growth factor [11] and the mature regions of other TGF-β family members [57]. There are two minima, the stronger at 218 nm and a second at 208 nm which are characteristic of β-sheet and α-helical structures respectively [69]. The absolute negative value at 200 nm implies a degree of intrinsic disorder exists in the structure [65], [70], which may be functionally significant due to the different roles suggested for the propeptide region of the myostatin precursor, requiring flexibility in the N-terminal domain. To our knowledge, this is the first structural analysis for any TGF-β family precursor protein. Quite a different profile is observed for the MstnPP soluble aggregates with the very strong minimum at 208 nm indicating that the secondary structure is primarily α-helical in nature. Broadening of the spectrum between 215–225 nm may arise from contributions of β-sheet and α-helix to the spectra. As the CD spectra of protofibrils of other amyloid-forming proteins is characterised by β-sheet absorption at 215–218 nm [61], [71], [72], it is likely that the MstnPP soluble aggregates represent an oligomeric intermediate state prior to protofibril formation rather than protofibrils. Incubation of the aggregates overnight at pH 5.3 and 60°C produces a CD spectrum dominated by β-sheet where the α-helix minimum at 208 nm is completely replaced by a minimum at 218 nm. TEM images of the same sample (Fig. 4) reveals there are no fibrils at this stage but the aggregates are more compact and have begun to aggregate into large arrays, which may represent the start of β-sheet stacking as amyloid protofibrils. The ellipticity for the spectrum is comparatively low, similar to that observed in the fibrillogenesis of Aβ [65]. This reduction of signal is most likely due to differential absorption flattening, a phenomenon often seen in the CD spectra of samples containing suspensions of solid-phase material [61] and causing both a decrease in intensity and red-shift of all minima. After one week of incubation the spectrum is similar except for flattening of the 190 nm peak. At this stage, fibrils are present as observed by TEM analysis of an aliquot of the same sample (Fig. 4). The further flattening of the spectrum over time is likely to be due to both an increase in β-sheet structure as well as differential absorption flattening [61], [69]. CD shows that the soluble aggregates differ dramatically in secondary structure compared to native MstnPP and that the aggregates may represent an α-helical-containing intermediate in the transition to protofibrils and fibrils rich in β-sheet structure.

### MstnPP Protofibrils and Fibrils Are Resistant to Proteolytic Digest by Trypsin

**Figure 6:**
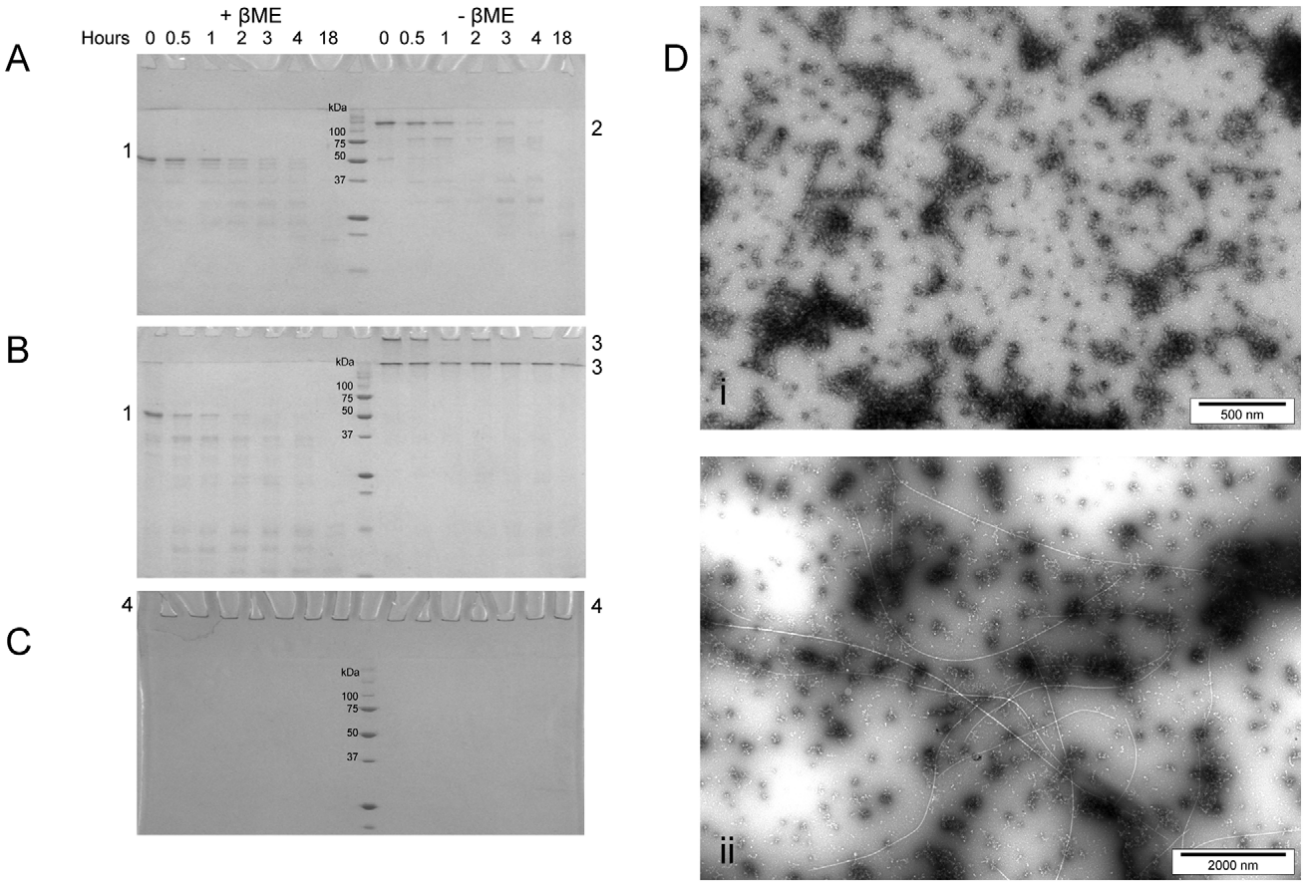
SDS-PAGE and TEM analysis showing MstnPP resistance to trypsin digestion. A. dimer; and B. soluble aggregates. Major bands are indicated: 1. MstnPP monomer; 2. MstnPP dimer; 3. MstnPP soluble aggregates. C. MstnPP fibrils: 4. prefibrillar aggregates plus fibrils. Samples were analysed in sample buffer +/− βME where βME concentration is 2 M. D. TEM shows resistance of (i) protofibrils and (ii) amyloid fibrils to trypsin. Trypsin digests were performed with a MstnPP∶trypsin ratio of 100∶1 (A and B) or 20∶1 (C and D) for 18 hours at 37°C.

Resistance to proteolytic digestion is another defining characteristic for the presence of amyloid fibrils [73], [74]. Comparative trypsin digestions were performed for the MstnPP fibril-containing sample, soluble aggregates and native dimer (Fig. 6). Digestion of the MstnPP dimer and aggregates was carried out with a MstnPP to trypsin ratio of 100∶1 (w/w) at 4°C, room temperature (approximately 22°C) and 37°C with samples taken after 0.5, 1, 2, 3, 4 and 18 hours. The precursor dimer and aggregates have comparable trypsin susceptibility at 37°C and are fully digested after an overnight incubation (18 hours, Fig. 6A and B respectively). The susceptibility of the soluble aggregates to trypsin digest suggests an open, flexible structure, consistent with CD results and supporting the conclusion that this species represents a prefibrillar intermediate rather than protofibrils. Prior to digestion the fibril-containing sample is not able to enter the top of the 4% stacking gel owing to its extremely large size. Following incubation with trypsin this property is maintained even after overnight incubation (18 h) at 37°C and a 5-fold increase in trypsin concentration (MstnPP∶trypsin 20∶1) (Fig. 6C). Inability to enter the stacking gel is not a definitive indication of proteolysis resistance since partial hydrolysis may result in products smaller than fibrils and/or protofibrils yet still large enough to be retained in the stacking gel. To address this possibility, TEM analysis revealed that both MstnPP protofibrils (Fig. 6Di) and mature fibrils (Fig. 6Dii) had unchanged morphology after trypsin incubation.

### MstnPP Aggregates Exhibit Amyloid Characteristics after Incubation at 37°C

**Figure 7:**
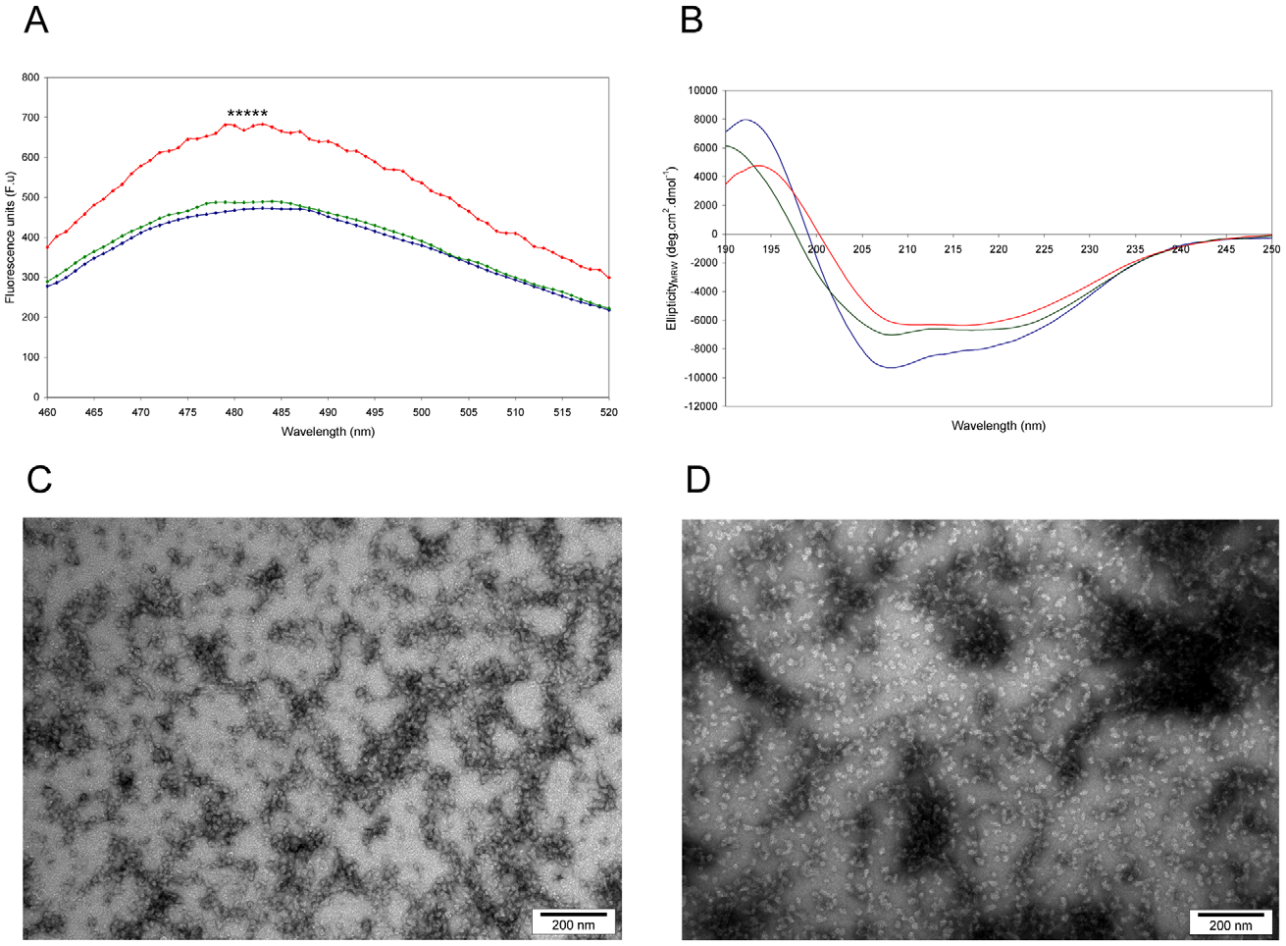
ThT binding, CD spectroscopy and TEM show the formation of prefibrillar structures and protofibrils by MstnPP aggregates at 37°C and pH 5.3. A. ThT binding assays before incubation (blue), after overnight incubation (green) and after one week incubation (red). ***** P<0.001 by Student's t-test using averaged values over peak. B. CD spectra of MstnPP soluble aggregates (blue), aggregates after overnight incubation at 37°C in pH 5.3 (green) and after one week incubation (red). C. TEM shows elongation and clustering after overnight incubation; and D. a granular to smooth transition and enhanced lateral association after one week.

The formation of amyloid fibrils by MstnPP at 60°C and pH 5.3 is comparable to conditions routinely used to promote rapid amyloid formation in *in vitro* model systems for amyloid diseases. To investigate the temperature range at which structural changes occur, MstnPP aggregates were analysed by CD from 10 to 65°C immediately after suspension in pH 5.3 (Fig. S3). The spectrum had changed significantly by 45°C with a loss of α-helix beginning from 30°C and the appearance of a characteristic amyloid β-sheet-rich spectrum at 65°C. Subsequently, MstnPP aggregates were incubated at 37°C at pH 5.3 and monitored with ThT binding, CD spectroscopy and TEM (Fig. 7). After overnight incubation ThT binding increased only slightly (Fig. 7A) and CD indicated that β-sheet aggregation has occurred (Fig. 7B). Under TEM, the 37°C protofibrils have a similar morphology to the 60°C samples, however aggregation is less extensive (Fig. 7C); elongation, clustering and ring-like structures are apparent. After one week of incubation at 37°C, ThT binding increased significantly (Fig. 7A) and CD showed a definite β-sheet transition (Fig. 7B). TEM images show increased aggregation, lateral association and a granular to smooth morphology transition (Fig. 7D). Although fibril formation did not occur at 37°C in the time period observed for incubation at 60°C, it is likely that the kinetics of fibril formation will be slower at the lower temperature. However, these results show that the properties of protofibrils, proposed to be direct precursors of mature amyloid fibrils, have been adopted at 37°C; amyloid formation is likely to occur over an extended period of time.

### MstnPP Aggregates Have a Negative Effect on C2C12 Myoblast Viability

**Figure 8:**
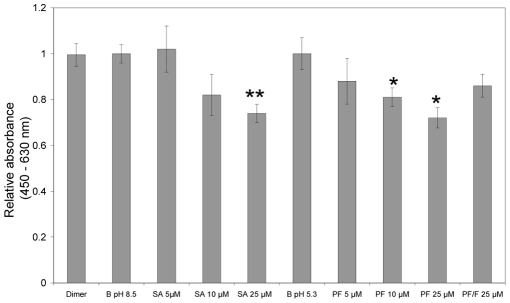
MstnPP aggregates and protofibrils (PF) are cytotoxic to C2C12 mouse myoblasts. C2C12 cytotoxicity assay using the WST-1 reagent where the difference in absorbance at 450 and 630 nm directly correlates to cell density after incubation with increasing concentrations of MstnPP soluble aggregates (SA, 5–25 µM), protofibrils (PF, 5–25 µM), 25 µM MstnPP dimer and 25 µM PF/F (fibrils). Concentrations are expressed as monomer equivalents. Cells incubated in media containing buffer (B) only (50 mM Tris-HCl pH 8.5, 150 mM NaCl for soluble aggregates and dimer; 0.005 mM HCl, pH 5.3 for protofibrils and fibrils) were used as a control. Error bars represent the standard error of the mean for triplicate samples from two independent experiments. Statistical significance was calculated using a paired Student's t-test where ** P<0.01 and * P<0.05.

Oligomeric species in the amyloid formation pathway of a number of proteins are cytotoxic when added to the media of cultured cells [41], [42], [65]. The effect of MstnPP soluble aggregates, protofibrils and fibrils on the viability of C2C12 mouse myoblasts was investigated by monitoring the absorbance of formazan (Fig. 8) produced after addition of WST-1 (Roche). Only viable cells reduce WST-1 to produce formazan with the ratio of absorbances at 450 and 630 nm correlated to the number of viable cells in the culture. C2C12 mouse myoblasts are the standard model cell-line used for the analysis of myostatin activity [16], [18]. Although human MstnPP is used here, sequence identity between the mouse and human myostatin precursors is 96%; the murine cell-line is therefore suitable for initial studies. 25 µM of soluble aggregates and at least 10 µM of protofibrils decreased cell viability significantly compared to buffer only and correctly folded MstnPP dimer controls, at the same pH. A solution containing a mixture of MstnPP protofibrils and fibrils, as well as lower concentrations of soluble aggregates and protofibrils, had a reduced effect. These results indicate that oligomeric species in the amyloid formation pathway of MstnPP affect the normal functioning of C2C12 myoblasts.

## Discussion

Amyloid formation is a predominant feature of sIBM [30] with the Alzheimer's disease protein Aβ associated with sIBM amyloid structures [31], [44]. A role for MstnPP in sIBM was proposed after co-localisation and direct association with Aβ was observed in diseased cells [31], and also because ER stress causes an upregulation of myostatin expression [45]. We now show that a human MstnPP misfolded species shares the morphological characteristics of amyloid protofibrils and the ability to bind the amyloid-specific dye thioflavin T. The α-helical secondary structure of these aggregates suggests they are most likely to represent an intermediate oligomeric species that occurs in the transition between native protein and β-sheet-rich protofibrils. When subjected to the mildly denaturing conditions of pH 5.3 and 60°C, the aggregates form amyloid protofibrils and after one week are able to form long, linear and unbranching amyloid fibrils. At 37°C, protofibrils and mature fibrils form over an increased time period. These *in vitro* results show that MstnPP is capable of amyloid protofibril and fibril formation, supporting a role for misfolding of the myostatin precursor in the pathogenesis of sIBM. MstnPP aggregates and protofibrils have a cytotoxic effect on mouse myoblasts when added to the culture medium.

CD spectroscopy shows there is a transition from the correctly folded protein structure to a structure consisting primarily of β-sheet. For a number of peptides and proteins this transition is from a loop or disordered region through an α-helical intermediate stage [36], [75]. Analysis of the differently folded forms of MstnPP suggests a shift towards a predominance of α-helix on misfolding. Whether this α-helical shift represents the intermediate stage described for other amyloid species is not known but is an attractive possibility. TEM images show a number of different morphological forms present in the aggregate solution. These may represent different oligomeric intermediates at different stages of the fibril formation process, some of which may be the direct precursors of protofibrils. Overnight incubation of MstnPP aggregates in pH 5.3 at 60°C produces a transition from the α-helical structure to one dominated by β-sheet. This result is consistent with the major models proposed for amyloid formation, which describe an unfolded state in equilibrium with a partly unfolded α-helix-containing intermediate that accelerates fibril formation [36], [37], [76]. Aggregation of the intermediate into oligomers precedes structural rearrangement into β-sheet-rich protofibrils. A detailed structure of protofibrils has not been determined; they are defined primarily by morphology, the ability to bind dyes such as ThT and a structural shift to a predominance of β-sheet. Amyloid fibrils have been well-studied structurally by NMR and X-ray fiber diffraction [77].

For growth factors such as myostatin, and a number of other secreted proteins, perhaps the biggest obstacle to refolding is the formation of the correct disulfide-bonded cysteine pairs in the ER [78]. Myostatin has the characteristic TGF-β cysteine knot motif in its mature domain [2], [10], [11] and so likely requires both an appropriate redox environment and the presence of disulfide-bond chaperones for correct folding in the ER. The myostatin propeptide that lies N-terminal to the mature domain has been proposed to have a chaperone-like function in the correct folding of the mature domain [8], [9]. During ER-stressed situations, such as an altered redox environment, misfolding can be extensive [48], [79] with the unfolded protein response (UPR) resulting in the elimination of misfolded proteins and/or apoptosis if necessary [48], [80], [81]. If the production of misfolded proteins overwhelms the UPR, protein aggregation may result in the generation of cytotoxic amyloid protofibrils [41], [42] and mature amyloid fibrils, contributing to amyloid diseases, including sIBM [32]. After refolding of MstnPP, misfolded yet soluble aggregates that form amyloid protofibrils and fibrils at a lowered pH can be observed. Although elevated temperature has been used to produce mature MstnPP fibrils quickly in this study, a physiological temperature allows protofibril and mature fibril formation over an extended time period; by comparison, fibril formation is expected to occur over many years *in vivo*.

sIBM is characterised by amyloid aggregation of the Aβ protein and severe muscle atrophy [30], [33]. Previous studies have shown that MstnPP associates with Aβ fibrils in diseased cells [31] and that ER stress results in upregulation of MstnPP in a cultured human sIBM-model [45]. Increased activity of the myostatin growth factor following MstnPP upregulation may contribute to atrophy in sIBM, however another study showed MstnPP accumulated with Aβ/AβPP within aggresomes following ER stress by proteasome inhibition in CHMFs [46]. This accumulation in aggresomes may prevent processing of MstnPP and decrease levels of circulating growth factor, as mature myostatin growth factor is absent in immunoblots of CHMFs transiently expressing AβPP [46].

The *in vitro* formation of MstnPP aggregates and amyloid species suggests a similar phenomenon could occur *in vivo* having a negative effect on cell viability and/or assisting in Aβ fibrillogenesis. The cytotoxicity exhibited by MstnPP aggregates and protofibrils in C2C12 cells supports a mechanism in which aggregation by MstnPP in sIBM contributes to muscle degeneration. This may occur independently of atrophy resulting from increased myostatin growth factor signalling. Even though MstnPP aggregates were localised to the cytoplasm, aggresomes and nuclear regions of sIBM tissue in previous studies [31], [46], an absence of extracellular MstnPP was not shown. Since MstnPP is secreted, possibly for local inhibition of muscle growth [14], post-secretion aggregation or secretion of aggregated/misfolded MstnPP may contribute to muscle fiber atrophy in sIBM via a cytotoxic mechanism that may involve alterations to endo- and exocytosis [65], alterations to Ca^2+^ homeostasis and the generation of reactive oxygen species [41]. The presence of pore-like structures under TEM suggests that the cytotoxic mechanism may include permeabilization of extracellular and/or intracellular membranes [43].

The results presented here show that human MstnPP is able to form cytotoxic amyloid-like aggregates and amyloid fibrils *in vitro*, raising the possibility that amyloid formation by MstnPP *in vivo* may contribute to the pathogenesis of sIBM.

## Materials and Methods

### MstnPP β-Aggregation Propensity by *In Silico* Analysis

MstnPP amino acids 21–375 were used in β-aggregation propensity prediction algorithms. Tango [52] and Waltz [55] output was reproduced from www.tango.embl.de; PASTA output was taken from www.protein.cribi.unipd.it/pasta [54]. Default settings were used for all programs.

### Expression of Recombinant MstnPP

Full-length human myostatin cDNA was used as a template for PCR cloning with primers encompassing cDNA corresponding to human MstnPP amino acid 21 (C-terminal to the signal peptidase cleavage site) to the C-terminal amino acid 375. PCR fragments were cloned into a modified pET vector via *BamHI* and *XhoI* restriction sites. Positive colonies were selected by colony PCR. The pET-MstnPP plasmid was isolated, sequenced and used to transform *E. coli* BL21(DE3) cells for protein expression. Transformed BL21 cells containing pET-MstnPP were grown in a starter culture of 20 mL LB in the presence of 100 µg/mL ampicillin (LB amp) at 37°C overnight. The starter culture was used to inoculate 2 L LB amp and cells were grown at 25°C to an OD_600_ of 0.8. Expression was induced by the addition of 0.1 mM IPTG and cells were incubated overnight (16–18 hours). Cells were collected by centrifugation at 4,000×*g* for 30 min at 4°C and stored at −20°C.

### Inclusion Body Isolation and Solubilisation and Refolding of Recombinant MstnPP

The frozen cell pellet from each 2 L culture was thawed at room temperature and resuspended in a final volume of 70 mL 20 mM Tris-HCl pH 8.5. Cells were lysed by two passages through a French Press at 5,000 psi and the inclusion body pellet was collected by centrifugation at 30,000×*g* at 4°C for 30 min. The inclusion bodies were washed with two 20 mL volumes of wash buffer (50 mM Tris HCl pH 8.5, 0.5 M NaCl, 10% glycerol, 0.5% Triton-X 100) and two washes with the same volume of milliQ H_2_O. Pellets were collected after each wash by centrifugation at 30,000×*g* for 10 min at 4°C. Solubilisation and refolding were carried out using methods published previously for the zebra-fish myostatin precursor protein [8] with some modifications. Each inclusion body pellet from 2 L of cell culture was solubilised in 8 mL 6 M guanidine hydrochloride (GndHCl), 50 mM Tris-HCl pH 8.5, 1 mM EDTA, 0.1 mM DTT, at room temperature, with shaking for 5 hours. Insoluble proteins and cell debris were removed by centrifugation at 17,000×g, 4°C for 30 min. The supernatant was acidified to pH ∼5.0 by adding a few drops of concentrated HCl and dialysed overnight against 6 M GndHCl, 50 mM MES pH 5.0, 1 mM EDTA to remove DTT. After dialysis, the sample was centrifuged again as above. The solubilised protein (8 mL) was rapidly diluted in 150 mL of freshly prepared refolding buffer, consisting of 50 mM Tris-HCl pH 8.5, 1 M NaCl, 0.5 M L-arginine, 5 mM EDTA, 5 mM reduced glutathione and 1 mM oxidised glutathione. The pH was re-adjusted to 8.5 before addition of protein. The protein was left to refold without agitation for 6 days at 4°C then centrifuged at 30,000×*g*, 4°C for 30 min before overnight dialysis into 20 mM Tris-HCl pH 8.5. Soluble protein was harvested as supernatant after a second centrifugation and filtered (0.45 µM).

### Purification of Refolded MstnPP Dimer and Soluble Aggregates

Purification of the *in vitro* refolded MstnPP dimer and misfolded soluble aggregates was performed by heparin affinity followed by gel filtration chromatography. The filtered protein solution was loaded onto a 5 mL HiTrap Heparin HP column (GE Healthcare) equilibrated with 50 mM Tris-HCl pH 8.5, at a flow rate of 1 mL/min. The flow-through containing unbound protein was discarded and a stepwise NaCl elution was used to elute the correctly folded dimer at 200 mM NaCl (H1) and the majority of soluble aggregates at 600 mM NaCl (H2). Elution was monitored by absorbance at 280 nm and differently folded forms were identified using non-reduced SDS-PAGE. Further purification of the MstnPP dimer was achieved using gel filtration chromatography. The 200 mM NaCl peak was filtered (0.22 µM), concentrated to 350 µL and applied to a Superdex S200 10/300 HR column (GE Healthcare) equilibrated with 50 mM Tris-HCl, pH 8.5, 150 mM NaCl, at a flow rate of 0.5 mL/min. The dimer eluted at approximately 13.5 mL (PD) and was separated from any remaining soluble aggregates that eluted in the void volume (PA).

### Western Blotting

Protein samples were separated on a 12% SDS-PAGE gel at 150 V and transferred to a nitrocellulose membrane overnight at 15 V (approximately 40–150 mA) in cooled Tris/glycine transfer buffer. The next day transfer of high molecular weight samples was ensured at 450 mA for one hour. Efficient transfer was confirmed by Ponceau S staining. Membranes were blocked with 5% non-fat milk in TBS-Tween (TBS-T; 20 mM Tris-HCl, pH 7.5, 150 mM NaCl, 0.1% Tween-20) for one hour at room temperature and subsequently incubated in rabbit anti-myostatin polyclonal primary antibody (Chemicon International), diluted 1/10,000 in 1% non-fat milk TBS-T, for 90 minutes. Following washing with TBS-T, membranes were incubated in a 1/50,000 dilution (1% non-fat milk, TBS-T) of horse radish peroxidase-conjugated anti-rabbit antibody (Jackson ImmunoResearch) for 90 minutes. All steps were performed at room temperature. After washing with TBS-T, proteins were visualised with the SuperSignal West Pico Chemiluminescent Substrate (Pierce) according to manufacturer's instructions.

### Generation of MstnPP Amyloid Fibrils

MstnPP soluble aggregates were sterile-filtered (0.22 µm), concentrated 10-fold and resuspended in dilute HCl solutions at a final concentration of 1 mg/mL, as measured by both Bradford assay and absorbance at 280 nm. Initially, a range of pH solutions were investigated for fibril formation from pH 1.6, as used for insulin amyloid formation [63] through to pH 6.3. Samples were placed at either 37°C or 60°C and monitored frequently for amyloid formation by Thioflavin T (ThT) fluorescence, TEM and CD spectroscopy. Subsequent to this optimisation, MstnPP aggregates were incubated at pH 5.3 (0.005 mM HCl) and 60°C for at least one week. Identical MstnPP amyloid fibrils were formed in equivalent solutions containing 0.1% sodium azide.

### Generation of Insulin Protofibrils and Fibrils

Insulin protofibrils and fibrils were produced as described [63]. In brief, different concentrations of insulin (bovine pancreas, Sigma) ranging from 0.5–2 mg/mL (85–340 µM) were prepared in 25 mM HCl (pH 1.6) and placed at 60°C for an hour (protofibrils) or at least overnight (fibrils) although incubation times were dependent on protein concentration.

### Negative-Stained Transmission Electron Microscopy

Dimer and aggregate samples direct from purification (heparin affinity or gel filtration) were buffer-exchanged into water. Amyloid solutions were used directly in dilute HCl. 200-mesh carbon-coated Formvar grids were placed on 30 µL protein solution (0.5 mg/mL) droplets for 45 seconds. Excess sample was drawn off using filter paper and the grids were placed on an equal volume of 2% uranyl acetate for 45 seconds. Excess stain was drawn off as above and the grids were air-dried briefly before viewing with a Philips CM10 transmission electron microscope. Fibril measurement and statistical analysis was performed using iTEM software (Olympus) with a sample size of 10 fibrils from the same grid.

### Thioflavin T Binding Assays

A solution of Thioflavin T (Sigma) was prepared at 400 uM in water [64] then diluted directly into the protein solution giving a final concentration of 20 µM per assay (as done previously for insulin [63]). A final protein concentration of 1 mg/mL was used for all samples. Experiments at pH 7.5 were performed after diluting both protein and ThT into 50 mM Tris-HCl pH 7.5, 100 mM NaCl. Fluorescence assays were conducted with the Perkin Elmer LS50B Luminescence Spectrometer with excitation at 450 nm and the emission spectrum measured from 460–530 nm. Error bars are standard errors for four independent measurements. The Student's t-test was performed using GraphPad Prism (GraphPad Software, Inc).

### Circular Dichroism Spectroscopy

CD spectra in the far-UV region (180–260 nm) were obtained on a Chirascan CD spectrometer (Applied Biophysics) using a 0.1 mm path cell and protein concentrations of 1 mg/mL at 4°C. For all samples, 20 runs were performed with 1 nm readings taken every 2.5 seconds, followed by smoothing and baseline subtraction. For CD thermal denaturation, 1 nm/2.5 second readings were taken at every 5°C increase in temperature from 10–65°C with a 30 second equilibration time at each temperature and a tolerance level of 0.2°C.

### Protease Resistance Analysis

MstnPP samples were tested for protease resistance using a myostatin to trypsin (bovine pancreas, Sigma) ratio of 100∶1 or 20∶1 (w/w). 100∶1 solutions were incubated at 4°C, room temperature or 37°C. Samples were taken at 0.5, 1, 2, 3, 4 and 18 hours (overnight), immediately denatured to end the reaction and analysed by reducing and non-reducing SDS-PAGE. For subsequent protease resistance analysis of MstnPP amyloid fibrils, the myostatin to trypsin ratio was 20∶1 (w/w) with incubation at 37°C for 4 and 18 hours and analysis by SDS-PAGE and TEM.

### Cell Culture and Incubation with Protein Aggregates

C2C12 mouse myoblasts were cultured in Advanced Dulbecco's Modified Eagle's Medium (Gibco, Invitrogen) containing 4.5 g/L D-glucose and 110 mg/L sodium pyruvate and supplemented with 10% foetal calf serum, 4 mM *L*-glutamine and penicillin/streptomycin. Cells were incubated in a 5% CO_2_ humidified environment at 37°C. Cells were plated in fresh medium in optical bottom 96-well plates (Nunc) at a density of 2,500 cells/well for cytotoxicity assays. After 24 hours, media was removed and 100 µL/well fresh medium containing protein was added. Two independent experiments with triplicate wells were used for each condition. Cells incubated in media containing buffer (B) only (50 mM Tris-HCl pH 8.5, 150 mM NaCl for soluble aggregates and dimer; 0.005 mM HCl, pH 5.3 for protofibrils and fibrils) were used as a control. Concentrations of myostatin samples used in the assay were comparable to those from previous protein fibril studies [41], [42], [65].

### WST-1 Colorimetric Assay for Cytotoxicity

The WST-1 reagent (Roche) is a tetrazolium-based salt that can be metabolized by viable cells only to produce a water-soluble formazan product measurable in a colorimetric assay. After a 24-hour incubation with protein, 10 µL WST-1 was added to each well and the plate was incubated for a further 3 hours. Absorbance at 450 nm was measured in a PowerWave XS 96-well plate reader (BioTek Instruments, Inc) with 630 nm readings taken as reference. Phenol red-free media was not used as the indicator does not interfere with the WST-1 assay; as phenol red absorbs slightly at 450 nm however, wells containing media only were used as an additional control [82].

## Supporting Information

Figure S1 *In silico* predictions of propensity for β-sheet aggregation (A and B) and regions responsible for amyloid formation (C) by MstnPP. (A) Tango; (B) PASTA; and (C) Waltz. The MstnPP amino acid sequence from residues 21–375 were used for the calculations at a theoretical pH of 7. Default settings were used for all algorithms.
(0.24 MB DOC)

Figure S2 Western blot and SDS-PAGE of MstnPP purification procedure. (A) 12% reducing (+ βME) vs non-reducing (− βME) SDS-PAGE and (B) subsequent Western blot of purification procedure. Lanes are as follows: H1, heparin peak 1; H2, heparin peak 2; PD, gel filtration purified dimer; PA, gel filtration purified aggregates. Major bands are indicated: 1. soluble aggregates; 2. dimer; 3. monomer. β-ME concentration in + β-ME lanes is 2 M.
(1.42 MB DOC)

Figure S3 CD thermal denaturation of MstnPP soluble aggregates at pH 5.3 shows a gradual transition from an α-helical dominated spectrum at 10°C to one rich in β-sheet at 65°C. Significant loss of the α-helical minimum begins at 40°C.
(0.21 MB DOC)
