# Supplementary material for: Cytotoxic Aggregation and Amyloid Formation by the Myostatin Precursor Protein
Source: PLoS One. 2010 Feb 11;5(2):e9170. doi: 10.1371/journal.pone.0009170 (PMC2820090; doi:10.1371/journal.pone.0009170)
Supplement: Figure S1 — In silico predictions of propensity for β-sheet aggregation (A and B) and regions responsible for amyloid formation (C) by MstnPP. (A) Tango; (B) PASTA; and (C) Waltz. The MstnPP amino acid sequence from residues 21–375 were used for the calculations at a theoretical pH of 7. Default settings were used for all algorithms. (0.24 MB DOC) [file pone.0009170.s001.doc]

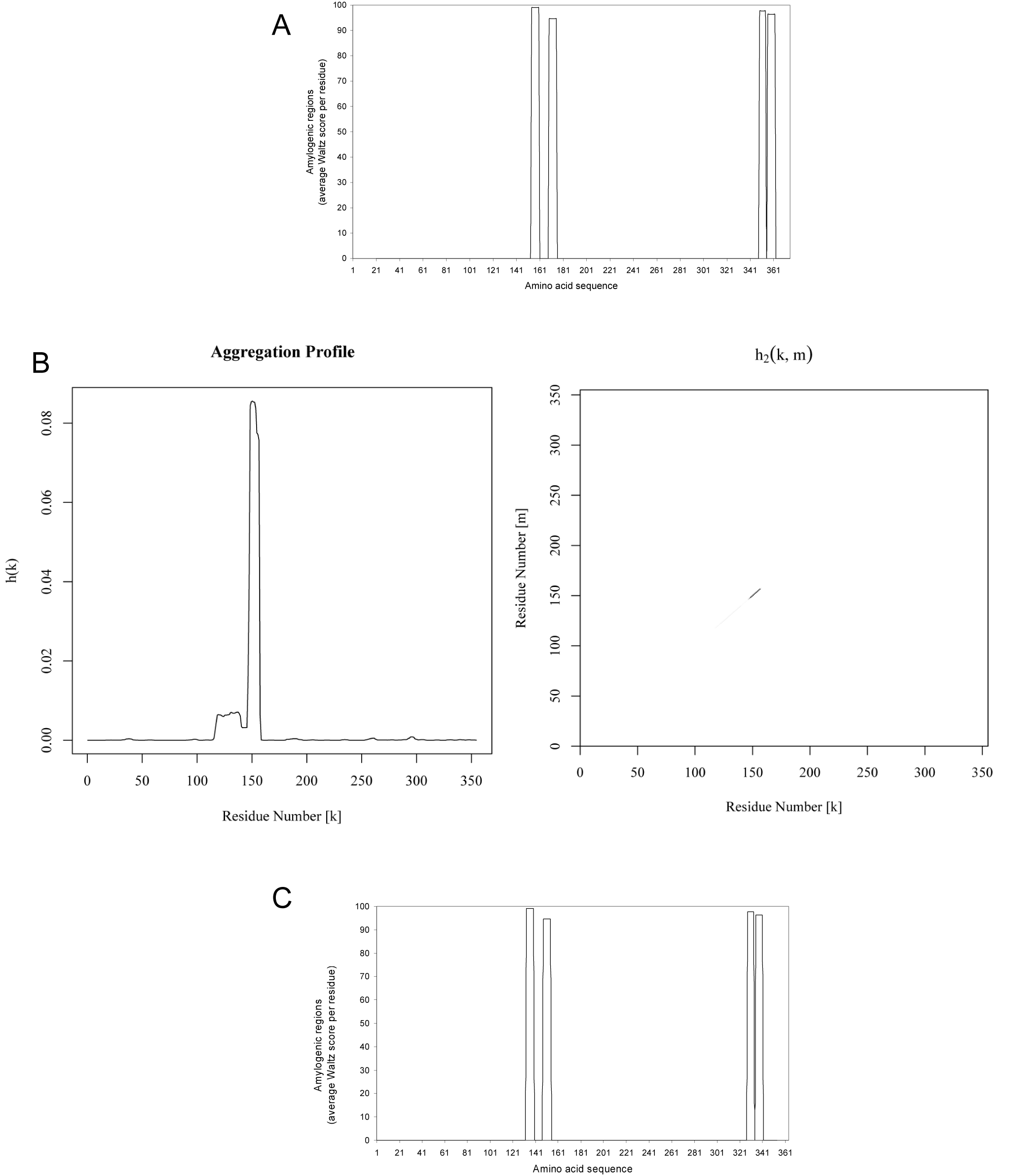


Figure S1: *In silico* predictions of propensity for β-sheet aggregation (a and b) and regions responsible for amyloid formation (c) by MstnPP. (a) Tango; (b) PASTA; and (c) Waltz. The MstnPP amino acid sequence from residues 21-375 were used for the calculations at a theoretical pH of 7. Default settings were used for all algorithms.
