# Supplementary material for: Cytotoxic Aggregation and Amyloid Formation by the Myostatin Precursor Protein
Source: PLoS One. 2010 Feb 11;5(2):e9170. doi: 10.1371/journal.pone.0009170 (PMC2820090; doi:10.1371/journal.pone.0009170)
Supplement: Figure S2 — Western blot and SDS-PAGE of MstnPP purification procedure. (A) 12% reducing (+ βME) vs non-reducing (− βME) SDS-PAGE and (B) subsequent Western blot of purification procedure. Lanes are as follows: H1, heparin peak 1; H2, heparin peak 2; PD, gel filtration purified dimer; PA, gel filtration purified aggregates. Major bands are indicated: 1. soluble aggregates; 2. dimer; 3. monomer. β-ME concentration in + β-ME lanes is 2 M. (1.42 MB DOC) [file pone.0009170.s002.doc]

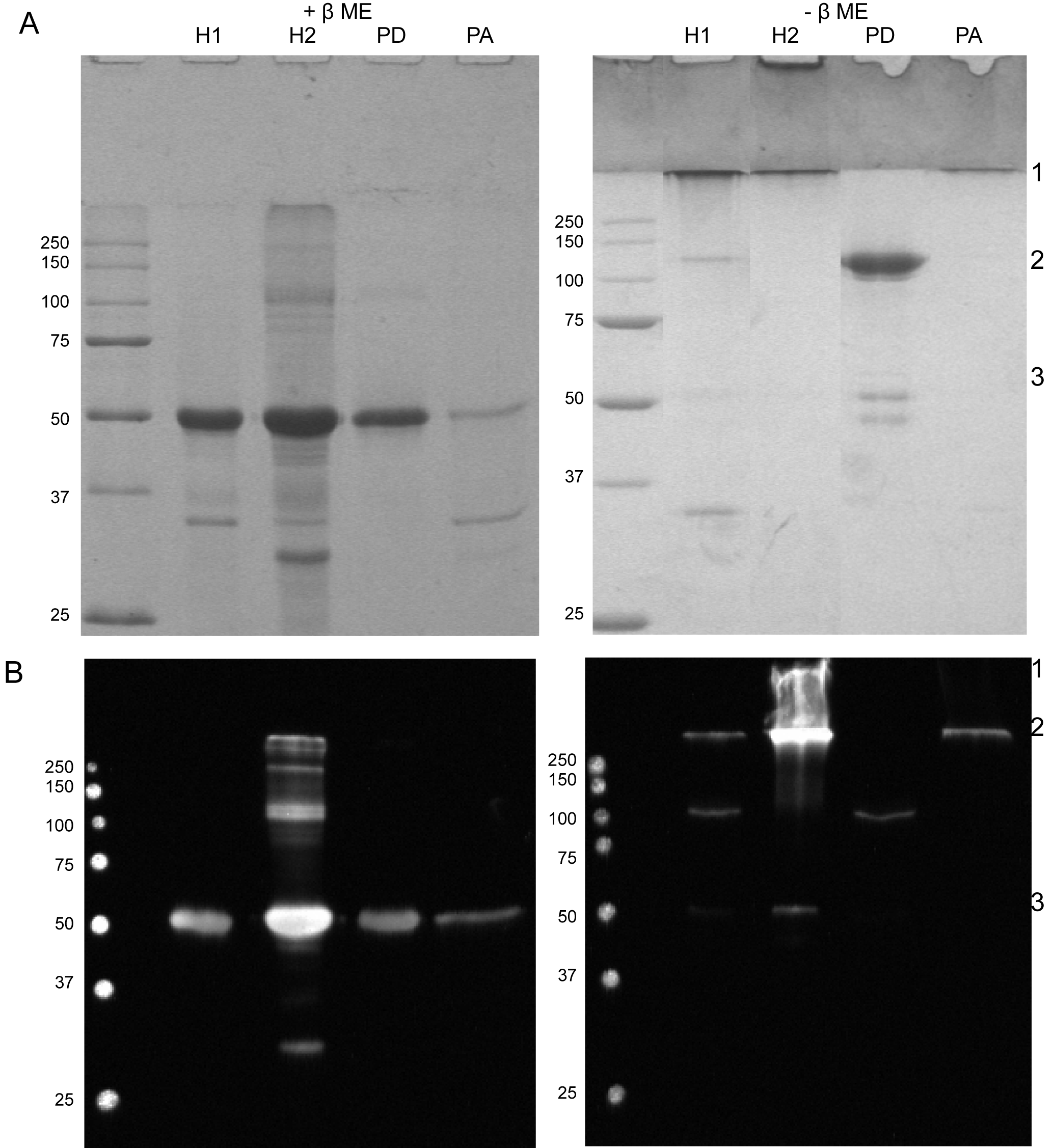


Figure S2: Western blot and SDS-PAGE of MstnPP purification procedure. (a) 12 % reducing (+ βME) vs non-reducing (- βME) SDS-PAGE and (b) subsequent Western blot of purification procedure. Lanes are as follows: H1, heparin peak 1; H2, heparin peak 2; PD, gel filtration purified dimer; PA, gel filtration purified aggregates. Major bands are indicated: 1. soluble aggregates; 2. dimer; 3. monomer. β-ME concentration in + β-ME lanes is 2 M.
