# Supplementary material for: Cytotoxic Aggregation and Amyloid Formation by the Myostatin Precursor Protein
Source: PLoS One. 2010 Feb 11;5(2):e9170. doi: 10.1371/journal.pone.0009170 (PMC2820090; doi:10.1371/journal.pone.0009170)
Supplement: Figure S3 — CD thermal denaturation of MstnPP soluble aggregates at pH 5.3 shows a gradual transition from an α-helical dominated spectrum at 10°C to one rich in β-sheet at 65°C. Significant loss of the α-helical minimum begins at 40°C. (0.21 MB DOC) [file pone.0009170.s003.doc]

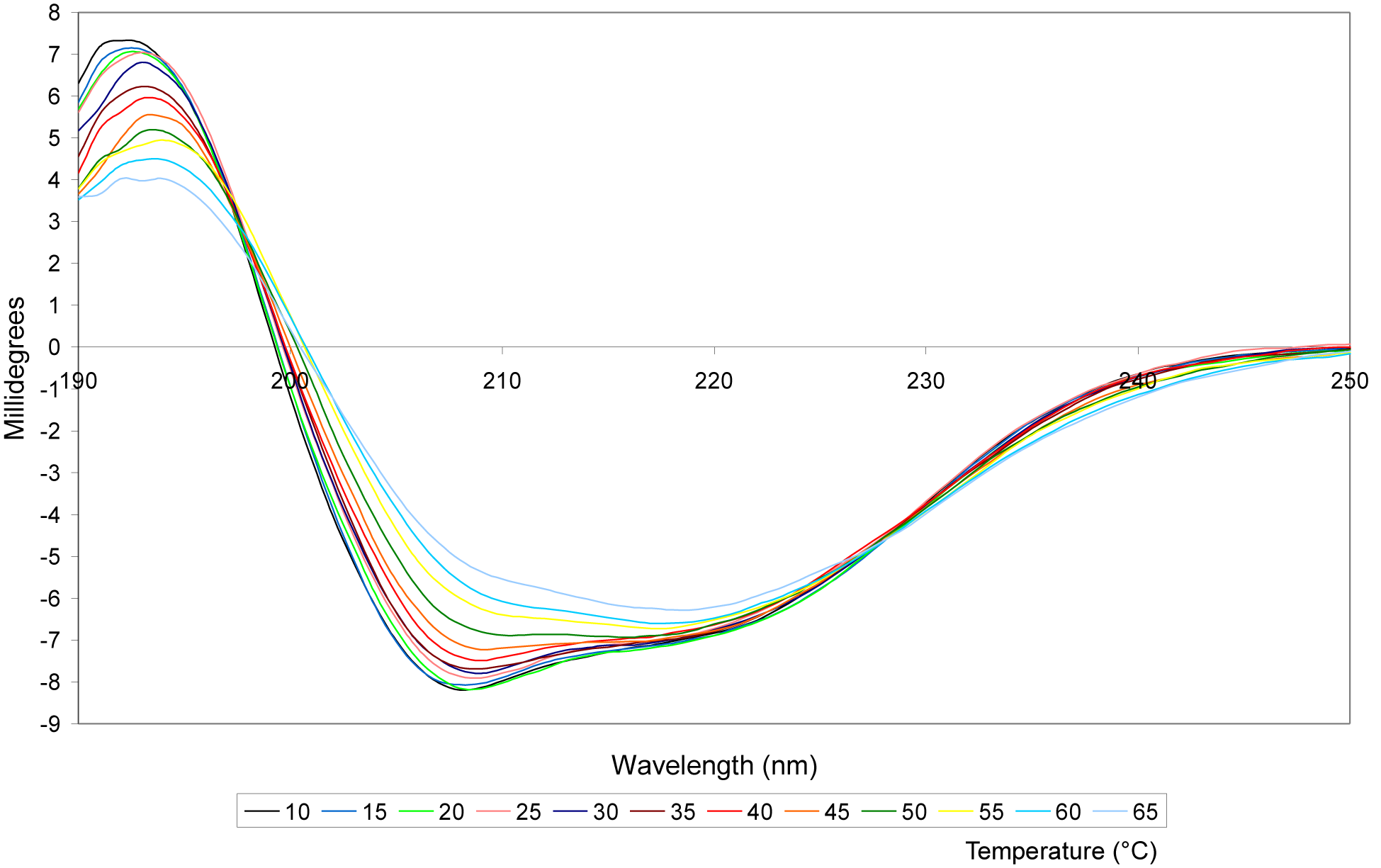


Figure S3: CD thermal denaturation of MstnPP soluble aggregates at pH 5.3 shows a gradual transition from an α-helical dominated spectrum at 10°C to one rich in β-sheet at 65°C. Significant loss of the α-helical minimum begins at 40°C.
